# Efficacy of probiotics combined with metformin and a calorie-restricted diet in obese patients with polycystic ovary syndrome

**DOI:** 10.12669/pjms.41.3.10554

**Published:** 2025-03

**Authors:** Jin Luo, Zhenyu Li, Zhibin Wang, Yashuang Ding, Peng Gao, Yuping Li

**Affiliations:** 1 Jin Luo Department of Gynecology, 73rd Army Group Military Hospital, Xiamen City, Fujian Province 361000, P.R. China; 2 Zhenyu Li Department of Rehabilitation, 73rd Army Group Military Hospital, Xiamen City, Fujian Province 361000, P.R. China; 3 Zhibin Wang Department of Medical Affair, 73rd Army Group Military Hospital, Xiamen City, Fujian Province 361000, P.R. China; 4 Yashuang Ding Department of Traditional Chinese Medicine, 73rd Army Group Military Hospital, Xiamen City, Fujian Province 361000, P.R. China; 5 Peng Gao Department of Medical Affair, 73rd Army Group Military Hospital, Xiamen City, Fujian Province 361000, P.R. China; 6 Yuping Li Department of Gynecology, 73rd Army Group Military Hospital, Xiamen City, Fujian Province 361000, P.R. China

**Keywords:** Calorie restricted diet, Obese patients, Polycystic ovary syndrome, Probiotics, Metformin

## Abstract

**Objective:**

To explore the efficacy of probiotics combined with metformin and a calorie-restricted diet in obese patients with polycystic ovary syndrome (PCOS).

**Methods:**

Clinical data of 141 obese PCOS patients treated in the 73rd Group Army Hospital from June 2021 to December 2023 were retrospectively analyzed. Patients were grouped according to the treatment records: metformin group (n=69), patients treated with metformin and a calorie-restricted diet) and combined group (n=72, patients treated with probiotics combined with metformin and a calorie-restricted diet). Levels of endocrine hormone indicators, Homeostasis Model Assessment of Insulin Resistance (HOMA-IR), Fasting blood glucose (FPG), gut microbiota status, and Body mass index (BMI) were compared before and after the treatment in two groups.

**Results:**

After the treatment, the levels of endocrine hormone indicators, HOMA-IR, and FPG in both groups were significantly reduced compared to pre-treatment levels and were significantly lower in the combined group compared to the metformin group (*P*<0.05). Abundance of gut microbiota and Shannon Wiener diversity index in both groups significantly increased after the treatment and were markedly higher in the combined group than in the metformin group (*P*<0.05). Treatment led to a significant reduction in the body mass index (BMI) in all patients (*P*<0.05). However, post-treatment BMI was comparable in the two groups (*P*>0.05).

**Conclusions:**

In obese patients with PCOS, adding probiotics to the metformin and calorie-restricted diet regimen is more effective in regulating hormone levels, improving blood sugar and insulin resistance, regulating gut microbiota status, and reducing BMI than metformin combined with calorie-restricted diet alone.

## INTRODUCTION

Polycystic ovary syndrome (PCOS) is common in women of childbearing age, with an incidence of about 8~13%.^1^ Obese patients account for about 30~70% of all PCOS cases,^2^ and generally have a higher risk of endocrine-related complications.^3^,^4^ Therefore, addressing obesity in PCOS patients is crucial to ensure a good prognosis.

The usual approach to weight management of patients with PCOS includes a combination of medications and nutritional support. Metformin, a commonly used drug that was shown to enhance insulin sensitivity and regulate blood sugar, is routinely used for the clinical treatment of obesity in women with PCOS.^5^ Additionally, as indicated by the Consensus of Medical Nutrition Treatment Experts on Overweight/Obesity in China, calorie-restricted energy-balanced diets have a positive impact on reducing weight in the obese population.^6^

However, due to the multifaceted nature of PCOS, it is still difficult to control the weight and clinical symptoms of obese patients with PCOS by simply limiting calorie intake and medication.^1^,^7^ With the current developments in the clinical research of PCOS, it has been found that the incidence and progression of obesity in PCOS are closely related to the status of gut microbiota, as obese PCOS patients often have a decrease in gut microbiota diversity, which not only has adverse effects on intestinal function but also directly affects their metabolism and immunity.^1^,^7^,^8^ Several studies have indicated that supplementing exogenous probiotics can help improve the therapeutic effect of standard treatment regimens in obese patients with PCOS.^9^ However, the research on probiotics combined with metformin based on calorie-restricted diets is still scarce.^10^ Therefore, this study aimed to evaluate the intervention value of probiotics combined with metformin and diet in obese patients with PCOS to provide guidance for healthcare professionals.

## METHODS

Clinical data of 141 obese PCOS patients treated in the 73rd Group Army Hospital from June 2021 to December 2023 were retrospectively selected. According to the method of treatment, patients were divided into a metformin group (n=69; treated with metformin and a calorie-restricted diet) and a combined group (n=72; treated with probiotics combined with metformin and a calorie-restricted diet).

### Ethical Approval:

The ethics committee of the 73^rd^ Group Army Hospital approved this study (No. LL20240501, Date: May 29^th^ 2024).

### Inclusion criteria:

- Female patients meeting 2003 Rotterdam criteria for PCOS.^11^
- Body mass index (BMI) ≥25 kg/m^2^.
- Women aged ≥ 20 years old.
- Completed three months of treatment.
- The clinical data is complete.

### Exclusion criteria:

- Patients with adrenal or ovarian tumors.
- Patients with functional impairments in organs such as kidneys and liver.
- Secondary obesity patients.
- Patients with genetic, metabolic, and endocrine system diseases.
- Patients with digestive system diseases.
- Patients took probiotics or prebiotics before the study.
- Breastfeeding and pregnant women.

### Treatment methods:

For all patients, a daily total energy intake of 25-30 kcal/kg was ensured, with carbohydrates (mainly starchy foods) providing 40% to 50% of the energy, protein function providing 15% to 20%, and fat providing 20% to 30% energy. Dietary fiber intake of 25-30 g/d was ensured, and monosaccharides/disaccharides and beverages were strictly limited.

### Metformin group:

Patients were treated with metformin and the calorie-restricted diet. Metformin (Shanghai Shiguibao Pharmaceutical Co., Ltd., specification: 0.5g/tablet), 0.5g orally was taken before bedtime every night in the first week, 0.5g orally with breakfast and before bedtime in the second week, and 0.5g orally with meals three times a day from a third week onwards. The length of the continuous treatment was three months.

### Combined group:

Patients treated with probiotics in addition to metformin and the calorie-restricted diet. The metformin regimen was the same as that of the metformin group. Bifidobacterium Triple Viable Capsules (Shanghai Shangyao Xinyi Pharmaceutical Co., Ltd., specification 0.21g/capsule) were orally taken at a dose of 0.84g/time, twice a day, continuously for three months.

### Observation indicators:

The indicators were recorded before and after three months of treatment.

- Basic information: age, course of the disease, BMI, waistline and hip measurements, and education level.
- Serum levels of estradiol (E2), testosterone (T), follicle-stimulating hormone (FSH), and luteinizing hormone (LH) were measured by radioimmunoassay using the reagent kit from Shanghai Xinfan Biotechnology Co., Ltd. before starting the treatment and after three months of therapy.
- Homeostasis Model Assessment of Insulin Resistance (HOMA-IR) was used to evaluate insulin resistance. It was calculated based on fasting blood glucose (FPG) levels, measured by a blood glucose meter, and fasting insulin (FINS), measured by radioimmunoassay. HOMA-IR: (FPG × FINS)/22.5.
- Intestinal microbiota status was determined by the α-diversity analysis (abundance and Shannon Wiener index) of the fecal samples.

### Statistical Analysis:

All data analyses were conducted using SPSS 25.0 software (IBM Corp, Armonk, NY, USA). The measurement data were represented by mean ± standard deviation, an independent sample t-test was used for inter-group comparison, and a paired t-test was used for intra-group before and after comparison. Count data was analyzed using the chi-square test to represent the number of cases. P<0.05 indicated a statistically significant difference.

## RESULTS

**Fig.1:**
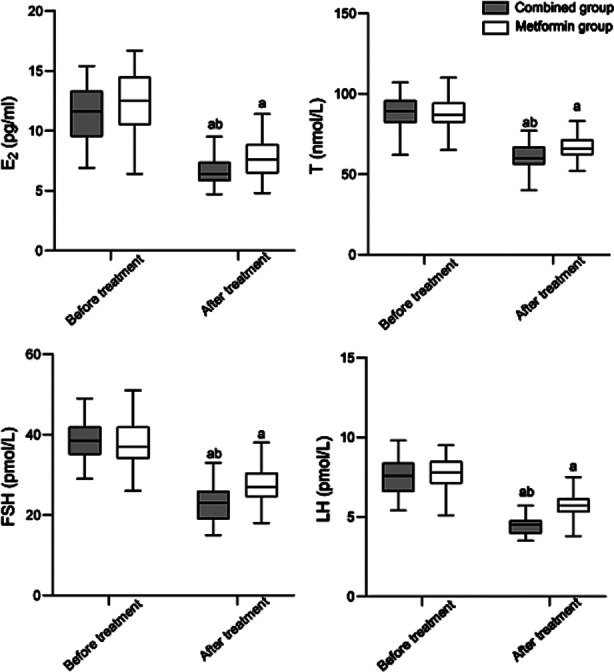
Comparison of endocrine hormone indicators between two groups. Estradiol (E2), testosterone (T), follicular stimulating hormone (FSH), and luteinizing hormone (LH); Compared with before treatment in the same group, ^a^*P*<0.05; Compared with the metformin group after treatment, ^b^*P*<0.05.

This study included 141 patients. Of them, 69 were in the metformin group, and 72 were in the combined group. There was no significant difference in baseline data between the groups (*P*>0.05) (Table-I). Before the treatment, the two groups had no significant difference in E2, T, FSH, and LH levels (*P*>0.05). After the treatment, levels of E2, T, FSH, and LH in both groups significantly decreased and were significantly lower in the combined group compared to the metformin group (*P*<0.05), Fig.1.

**Table-I:** Comparison of baseline data between two groups.

Baseline data	Combined group (n=72)	Metformin group (n=69)	t/χ^2^	P
Age (year)	31.53±5.52	30.67±5.867	0.898	0.371
Course of disease (year)	2.81±1.06	2.99±1.16	-0.965	0.336
BMI (kg/m^2^)	29.56±2.83	30.00±3.19	-0.869	0.387
Waistline (cm)	77.85±7.84	78.45±8.81	-0.429	0.669
Hip (cm)	92.44±8.28	93.80±9.1275	-0.922	0.358
Education level (n)				
Junior high school and below	30 (41.67)	23 (33.33)	1.043	0.307
High school and above	42 (58.33)	46 (66.67)		

**Fig.2:**
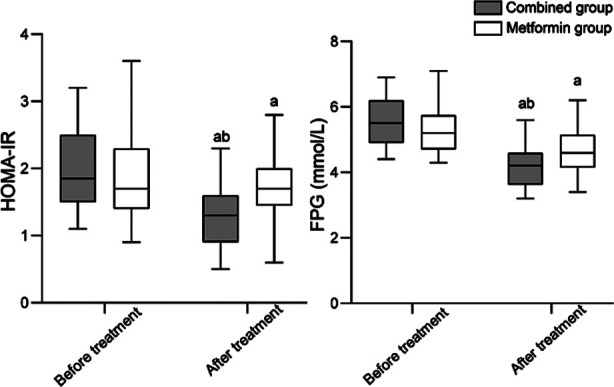
Comparison of HOMA-IR and FPG levels between two groups. Homeostasis Model Assessment of Insulin Resistance (HOMA-IR), fasting plasma glucose (FPG); Compared with before treatment in the same group, ^a^*P*<0.05; Compared with the metformin group after treatment, ^b^*P*<0.05.

**Fig.3:**
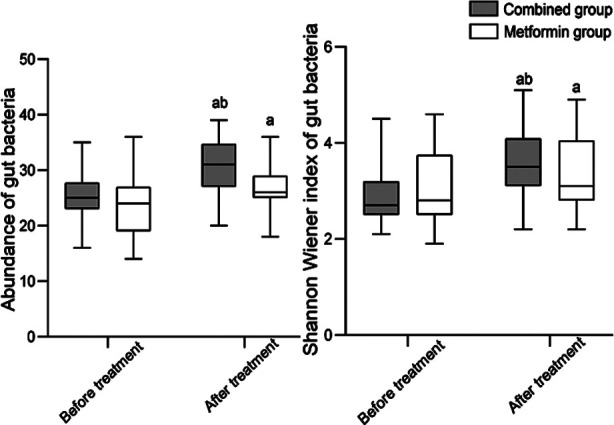
Comparison of gut microbiota status between two groups. Compared with before treatment in the same group, ^a^*P*<0.05; Compared with the metformin group after treatment, ^b^*P*<0.05.

**Fig.4:**
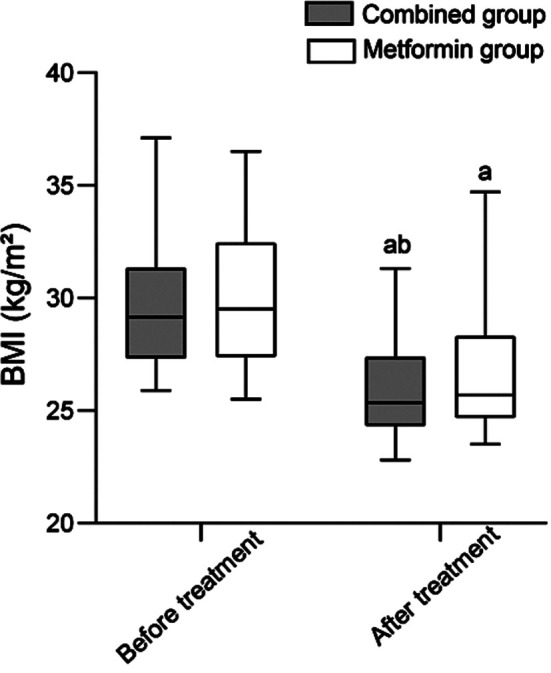
Comparison of BMI levels between two groups. Compared with before treatment in the same group, ^a^*P*<0.05; Compared with the metformin group after treatment, ^b^*P*<0.05.

Before the treatment, HOMA-IR and FPG levels were comparable in the two groups (*P*>0.05). Treatment significantly lowered both indexes in all patients. Post-treatment values of HOMA-IR and FPG in the combined group were significantly lower than in the metformin group (*P*<0.05), Fig.2. Before the treatment, there was no significant difference in the abundance of gut microbiota and Shannon Wiener index between the two groups (*P*>0.05). After the treatment, the abundance of gut microbiota and Shannon Wiener index in both groups significantly increased compared to pre-treatment levels and was markedly higher in the combined group than in the metformin group (*P*<0.05), Fig.3. After the treatment, BMI of patients in both groups significantly decreased (*P*<0.05). The two groups had no significant difference in the post-treatment BMI (*P*>0.05), Fig.4.

## DISCUSSION

The results of this study show that probiotics combined with metformin and a calorie-restricted diet can more effectively regulate the levels of endocrine hormone indicators and improve blood sugar and insulin resistance in obese patients with PCOS compared to metformin and diet alone. In addition to routine interventions such as improving insulin resistance and regulating menstrual cycles, obese patients with PCOS also require dietary management to control their weight effectively.^12^,^13^ Zhang et al.^14^ found that limiting calorie intake can effectively regulate blood sugar and body composition in patients with PCOS and improve hormone levels, allowing patients to reach weight management goals. Deshmukh et al.^15^ also explored the value of calorie-restricted diets for obese patients with PCOS and showed that dietary management was associated with improved androgen index, metabolic indicators, and body composition and a significant reduction in the waist-to-hip ratio. However, while lifestyle behavior intervention plays an important role in weight loss and delaying the progression of PCOS, the intervention effect is easily affected by patient compliance, and the intervention period is extended. Therefore, combining dietary changes with therapeutic drugs may improve the overall effect of the treatment and effectively regulate abnormal glucose and lipid metabolism.^16^,^17^

In agreement with previous reports, the results of the present study showed that the levels of endocrine hormone indicators, HOMA-IR, and FPG in the metformin group improved after the treatment. However, while metformin is suitable for the treatment of PCOS, studies have shown that adding metformin to a low-calorie diet does not significantly improve serum glucose or insulin concentration and insulin resistance in women with PCOS.^18^,^19^

It has been demonstrated that the combination of probiotics and metformin treatment on a calorie-restricted diet resulted in better improvement in endocrine hormones, HOMA-IR, FPG, and gut microbiota in patients compared to the metformin and diet alone. The study by Ji X et al.^10^ also showed that in patients with PCOS, the combination of probiotics and metformin treatment resulted in significantly lower BMI, fasting blood glucose, HOMA-IR, and lipid levels compared to the probiotics alone or metformin groups. Metformin can inhibit fat breakdown, enhance anaerobic glycolysis of glucose, and effectively inhibit glycogen output.^17^,^19^ Furthermore, metformin can effectively prevent insulin resistance, reduce the synthesis rate of triglycerides, significantly lower blood sugar levels, and reduce visceral fat accumulation, thereby reducing weight.^20^,^21^ Patients in the present study used metformin combined with Bifidobacterium triple viable capsules for the treatment. The main components of the probiotic capsules used in this study are Bifidobacterium, Lactobacillus acidophilus, and Enterococcus faecalis, which can improve the proportion of beneficial bacteria in the intestine, effectively inhibit the action of some pathogenic bacteria, maintain the integrity of intestinal mucosa and ensure normal intestinal motility.^21^ Probiotics also inhibit harmful bacteria from producing endotoxins and maintain normal physiological functions of the human gut. Gut microbiota is closely related to obesity and glucose and lipid metabolism disorders, and orally taken probiotics can correct the abnormal state of gut microbiota in obese patients with PCOS, thereby playing a therapeutic role.^20^-^22^

The present study has important clinical implications. It has demonstrated that regulating intestinal flora as a therapeutic target and selecting oral probiotics for treatment can maintain the stability of the intestinal environment of obese patients with PCOS, effectively protect the intestinal mucosa, and play a vital role in regulating metabolic abnormalities. The results of this study may be used to improve current methods of managing obese patients with PCOS, as adding a probiotic treatment to the routine metformin regimen is a safe, cost-efficient, and effective way of improving symptoms in this group of patients.

### Limitations:

Firstly, it is a single-center retrospective analysis with a small sample size, which has a certain risk of bias in sample selection. Secondly, after regular treatment, patients did not undergo long-term follow-up on their daily scheduled medication. Although emphasizing its importance to patients, it cannot be ruled out that the final efficacy may have been reduced due to patient compliance. Thirdly, international guidelines now recognize that weight loss and maintenance in obesity require lifestyle and medication treatment.

### Recommendations:

Future larger-scale studies with longer follow-ups are needed to understand better the role of probiotics combined with metformin in managing PCOS based on calorie-restricted diets.

## CONCLUSION

The addition of probiotics to the standard regimen of metformin and a calorie-restricted diet can more effectively regulate the levels of endocrine hormone indicators, improve blood sugar and insulin resistance, regulate gut microbiota status, and help control BMI in obese patients with PCOS.

### Authors’ Contributions:

**JL:** Conceived and designed the study. Literature search.

**ZL**, **ZW**, **YD**, **PG** and **YL:** Collected the data and performed the analysis. Critical Review.

**JL:** Was involved in the writing of the manuscript and is responsible for the integrity of the study.

All authors have read and approved the final manuscript.
